# Retraction: A Femtomol Range FRET Biosensor Reports Exceedingly Low Levels of Cell Surface Furin: Implications for the Processing of Anthrax Protective Antigen

**DOI:** 10.1371/journal.pone.0269294

**Published:** 2022-05-26

**Authors:** The PLOS ONE Editors

Following the publication of this article [1], concerns were raised regarding the results presented in multiple figures. Specifically,

- The following data appear similar despite representing different conditions:
  ○ Fig 7 (right panel), lanes 1 and 3.
  ○ Fig 10A, lanes 2 and 4, and lanes 5 and 7 flipped horizontally.
- There appear to be vertical discontinuities in the following figures when colour levels are adjusted:
  ○ Fig 1, between lanes 1–2.
  ○ Fig 3 (lower left panel), between lanes 1–2.
  ○ Fig 7 (right panel), between lanes 2–3, 3–4 and 6–7.
  ○ Fig 9 (right panel), between lanes 1–2.
  ○ Fig 10A, between lanes 4–5.

The corresponding author stated that the raw image data underlying the above figures are no longer available. They acknowledged that the presented results are merged from multiple experiments, and stated that splice lines are clearly visible and were intentional.

In relation to the concerns in Figs 7 and 10, the corresponding author acknowledged that the data in the lanes listed above appear similar, but asserted that they are not identical. In the absence of raw image data, the editors remain concerned about the integrity of these figures.

In light of the concerns affecting multiple figure panels that question the integrity of these data, the *PLOS ONE* Editors retract this article.

AYS did not agree with the retraction and stands by the article’s findings. All other authors either did not respond directly or could not be reached.
